# Montelukast attenuates abdominal aortic aneurysm in rats: Anti-inflammatory and antioxidant effects

**DOI:** 10.1016/j.jvssci.2025.100405

**Published:** 2025-12-17

**Authors:** Gözde Tekin, Özge Çevik, Şule Çetinel, Göksel Şener, Mehmet Kızılay

**Affiliations:** a Department of Cardiovascular Surgery, Siyami Ersek Chest and Cardiovascular Surgery Training and Research Hospital, İstanbul, Turkey; b Department of Biochemistry, Faculty of Pharmacy, Adnan Menderes University, Aydın, Turkey; c Department of Histology and Embryology, Faculty of Medicine, Marmara University, Istanbul, Turkey; d Department of Pharmacology, Faculty of Pharmacy, Fenerbahçe University, Istanbul, Turkey

**Keywords:** Abdominal aortic aneurysm, Matrix metalloproteinase, Oxidative injury, Montelukast

## Abstract

**Objective:**

Oxidative stress and inflammation are widely recognized as central mechanisms in the pathogenesis of abdominal aortic aneurysm. This study sought to examine the potential protective properties of montelukast in a rat model of aortic aneurysm.

**Methods:**

Male Sprague-Dawley rats were randomly allocated into three experimental groups. Abdominal aortic aneurysm was induced using the calcium chloride (CaCl_2_) model, in which gauze soaked in 0.5 M CaCl_2_ was placed directly onto the adventitial surface of the infrarenal abdominal aorta for 15 minutes. After induction, the treatment group received daily intraperitoneal injections of montelukast (10 mg/kg) for 4 consecutive weeks. At the study end point, animals were euthanized, and infrarenal aortic tissues were harvested for biochemical and histological evaluations. Measured parameters included matrix metalloproteinase (MMP)-2 and MMP-9 expression, myeloperoxidase (MPO) activity, and 8-hydroxy-2′-deoxyguanosine levels. Antioxidant capacity was assessed through superoxide dismutase (SOD) activity assays. Histopathological examinations were performed, and statistical analysis was conducted using GraphPad Prism v.5.

**Results:**

Exposure to CaCl_2_ triggered pronounced oxidative injury and inflammation, as evidenced by elevated 8-hydroxy-2′-deoxyguanosine levels, increased MPO activity, reduced SOD activity, and upregulated MMP-2 and MMP-9 expression. Montelukast administration markedly attenuated these changes, normalizing oxidative and inflammatory markers while improving histopathological architecture.

**Conclusions:**

Montelukast effectively counteracted CaCl_2_-induced aortic damage. The protective effects of montelukast appear to be mediated through suppression of MMP activity, restoration of SOD levels, and reduction of MPO-driven oxidative injury. By mitigating both inflammatory and oxidative mechanisms, montelukast contributes to the preservation of aortic wall structure.

**Clinical Relevance:**

Abdominal aortic aneurysm remains a major vascular disorder without an effective pharmacological therapy to slow its progression. In this experimental study, montelukast, a leukotriene receptor antagonist widely used in asthma, attenuated abdominal aortic aneurysm formation in rats and was associated with increased superoxide dismutase activity, reduced myeloperoxidase levels, and suppressed matrix metalloproteinase activation. These combined antioxidant, anti-inflammatory, and matrix-stabilizing effects preserved aortic wall integrity. Given montelukast's established safety and clinical availability, these findings support its potential for future clinical investigation as a pharmacological approach to limit aneurysm progression.

**Article Highlights**

• **Type of Research:** Experimental in vivo study using a calcium chloride-induced rat model of abdominal aortic aneurysm
• **Key Findings:** Montelukast treatment significantly reduced aortic diameter expansion (*P* < .01) and matrix metalloproteinase-2/matrix metalloproteinase-9 expression (*P* < .05), decreased myeloperoxidase activity (*P* < .01), and restored superoxide dismutase levels (*P* < .05) compared with untreated abdominal aortic aneurysm rats. Histological evaluation confirmed reduced leukocyte infiltration and better preservation of elastic and collagen fibers.
• **Take Home Message:** In this rat model, montelukast, a leukotriene receptor antagonist with a well-established safety profile, was associated with reduced aneurysm progression and with biochemical and histological changes indicative of enhanced antioxidant and anti-inflammatory activity, including increased superoxide dismutase levels, decreased myeloperoxidase activity, and reduced matrix metalloproteinase expression.

Abdominal aortic aneurysm (AAA) is a progressive vascular disorder characterized by degradation of the aortic wall's structural integrity, leading to dilation and rupture with high mortality.^1^ Despite advances in surgical treatment, no effective pharmacological therapy has yet been established to prevent aneurysm formation or progression. Increasing evidence implicates oxidative stress and inflammation as key drivers of AAA pathogenesis.^2^^,^^3^

Excessive generation of reactive oxygen species (ROS) disrupts extracellular matrix (ECM) homeostasis, promotes proteolytic activity, and amplifies inflammatory cascades, resulting in medial degeneration. Among the proteolytic enzymes involved, matrix metalloproteinases (MMPs), particularly MMP-2 and MMP-9, play a central role in ECM degradation and vessel wall weakening,^4^, ^5^, ^6^ as shown in both experimental and human studies.^7^^,^^8^

Myeloperoxidase (MPO), a neutrophil-derived enzyme, contributes to oxidative injury by generating hypochlorous acid and other oxidants that further activate MMPs and damage vascular components.^9^^,^^10^ Conversely, antioxidant enzymes such as superoxide dismutase (SOD) mitigate ROS toxicity, and their depletion has been associated with aneurysm formation. Thus, therapeutic agents capable of decreasing oxidative stress and inflammation may hold promise in AAA management.^11^

From a therapeutic standpoint, medical interventions for AAA primarily aim to slow aneurysm growth and decrease the risk of rupture. Although drugs such as β-blockers, angiotensin-converting enzyme inhibitors, and statins have shown no significant effect on aneurysm diameter, they may decrease rupture risk. Given the established role of inflammation in AAA pathogenesis, anti-inflammatory agents have gained attention as potential therapeutic options.^12^

Leukotrienes (LTs), lipid mediators produced via the 5-lipoxygenase pathway, have been implicated in vascular inflammation and remodeling. Cysteinyl LTs (CysLTs) enhance vascular permeability and macrophage recruitment, leading to the upregulation of cytokines, MMPs, and ROS within the aortic wall.^13^^,^^14^

Montelukast, a selective CysLT_1_ receptor antagonist widely used in asthma, exhibits anti-inflammatory and antioxidant effects and has been shown to attenuate aortic aneurysm formation in mice and other experimental models.^15^^,^^16^

The present study aimed to evaluate whether montelukast exerts similar protective effects in a rat model of AAA induced by periaortic calcium chloride (CaCl_2_) application, focusing on its impact on MMP expression, MPO activity, and antioxidant defense mechanisms.^17^

## Methods

### Experimental groups

This study was approved by the Marmara University Local Ethics Committee for Animal Experiments (Approval No: 27.2015.mar) and conducted in accordance with the National Institutes of Health Guide for the Care and Use of Laboratory Animals and the ARRIVE guidelines, ensuring compliance with all ethical standards for animal experimentation. This work was supported by BAPKO (Coordination Unit for Scientific Research Projects)

Male Sprague-Dawley rats (300-350 g) were obtained from the Marmara University Animal Center. Animals were housed in a temperature-controlled facility (22 ± 2 °C) with 65% to 70% relative humidity and a 12-hour light/dark cycle, with free access to food and water. All experimental procedures complied with the Marmara University Animal Care and Use Committee guidelines.

The rats were randomly assigned to one of three groups (n = 10 per group):

1. Sham-operated control group (C),
2. AAA group (AA), or
3. AA + montelukast group.

The montelukast dosage was selected based on prior studies demonstrating its anti-inflammatory effects.^15^ Montelukast was freshly prepared each day and administered intraperitoneally at 10 mg/kg once daily at 18:00. The control and AA groups received the vehicle (1% ethanol in 1 mL saline/kg) under the same schedule for 2 weeks after sham surgery or AAA induction, respectively.

### Induction of AAA

AAA was induced using the established CaCl_2_ periaortic application model. Rats were anesthetized with ketamine (100 mg/kg) and chlorpromazine (30-50 mg/kg) and positioned supine for midline laparotomy. The intestines were gently displaced to the right, and the infrarenal abdominal aorta was exposed via blunt dissection. Baseline aortic diameter was measured before induction.

A piece of gauze saturated with 0.5 M CaCl_2_ was applied to the adventitial surface of the exposed aorta for 15 minutes. The abdominal cavity was then irrigated with warm sterile saline, and the incision was closed using 2/0 silk sutures. Postoperatively, the treatment group received montelukast as described for 4 consecutive weeks. Sham-operated control rats underwent laparotomy and exposure of the infrarenal abdominal aorta without CaCl_2_ application.

At the end of the study period, rats were euthanized by decapitation, and infrarenal aortic tissue samples were harvested for analysis. Before excision, postmortem microscopic images of the aorta were obtained to determine maximal aortic dilatation. A 26G needle (0.45 mm diameter) served as a calibration reference for image analysis, which was performed using ImageJ (National Institutes of Health). Measurements were made blindly by two independent observers, each performing three replicates, and the mean value was calculated for each sample.

### Western blot analysis for MMP-2 and MMP-9

Protein expression of MMP-2 and MMP-9 in aortic tissues was determined via Western blotting. Tissue homogenates were prepared in cell lysis buffer, and total protein concentration was measured using the Bradford assay.^18^ Equal amounts of protein were separated on 4% to 12% SDS-PAGE gels and transferred to polyvinylidene fluoride membranes. Membranes were blocked with bovine serum albumin and incubated overnight at 4 °C with primary antibodies against MMP-2, MMP-9, and β-actin (Santa Cruz Biotechnology). After TBST washes, membranes were incubated with horseradish peroxidase-conjugated secondary antibodies for 2 hours at room temperature. Protein bands were visualized using chemiluminescent substrates and quantified using ImageJ. Because the antibodies detect both pro- and active forms of MMP-2 and MMP-9, densitometric analysis was performed on the active forms, while pro-MMP bands were included as supportive indicators of overall MMP expression. All expression levels were normalized to β-actin.

### Measurement of MPO activity

MPO activity in aortic tissue was assessed according to the method of Hillegas et al.^19^ One unit of MPO activity was defined as the amount of enzyme that produced a change in absorbance at 460 nm over 3 minutes. Results were expressed as units per gram of tissue.

### Determination of 8-hydroxy-2′-deoxyguanosine levels

Genomic DNA was extracted from freshly collected aortic tissue using a commercial kit (Invitrogen). Tissue 8-hydroxy-2′-deoxyguanosine (8-OHdG) levels, an index of oxidative DNA damage, were quantified using a competitive ELISA kit (OxiSelect Oxidative DNA Damage ELISA, Cell Biolabs) in accordance with the manufacturer's instructions.

### Measurement of SOD activity

SOD activity was determined following the method of Mylroie et al^20^ and expressed as units per milligram of protein.

### Histological examination

For light microscopy, aortic tissue segments were fixed in 10% formaldehyde, dehydrated in graded ethanol, cleared in toluene, and embedded in paraffin. Sections (5 μm) were stained with hematoxylin and eosin and examined using an Olympus BX51 photomicroscope.

Histological features were evaluated semiquantitatively using a standardized scoring system commonly applied in vascular injury and aneurysm models. The following parameters were scored on a 0 to 3 scale:

• Leukocyte infiltration (0 = none, 1 = mild, 2 = moderate, 3 = severe)
• Elastic lamina disruption (0 = intact, 1 = mild fragmentation, 2 = moderate fragmentation, 3 = severe destruction)
• Collagen fiber integrity (0 = preserved, 1 = mildly disrupted, 2 = moderately disrupted, 3 = markedly disrupted)

A total histological damage score was calculated as the sum of these individual parameters for each section.

All slides were evaluated by two independent histologists who were masked to group allocation. Each observer scored the sections separately, and the mean value was used for analysis.

### Statistical analysis

Statistical evaluations were performed using GraphPad Prism v.5 (GraphPad Software). Data are presented as mean ± standard error of the mean. Biochemical data were compared using one-way analysis of variance followed by Tukey's post hoc test. Histological scores were analyzed using the Mann-Whitney *U* test. A *P* value of <.05 was considered statistically significant.

## Results

### Aortic diameter

The maximal infrarenal aortic diameter was measured, and the percentage of dilation was calculated according to the formula described by Morimoto et al^21^:

Dilationratio(%)=maximalaneurysmdiameter/nativeaorticdiameter×100

At baseline, all groups had similar aortic diameters. By week 4, the AA group exhibited a marked increase in aortic diameter compared with the sham group (*P* < .001). Montelukast treatment significantly reduced aortic dilation compared with the untreated AA group (*P* < .01) (Table).

**Table:** Dilatation ratio (%) for abdominal aortic aneurysm (*AA*) and montelukast-treated groups

Group	At the beginning	4 Weeks
Sham	100	118.2 ± 7.5
AA	100	173.4 ± 3.2^a^
AA + montelukast	100	141.2 ± 1.8^b^

a *P* < .001: AA vs sham (obtained at the same time point).

b *P* < .01: AA vs AA + montelukast group (obtained at the same time point).

### Western blot analysis of MMP2 and MMP9

**Fig 1:**
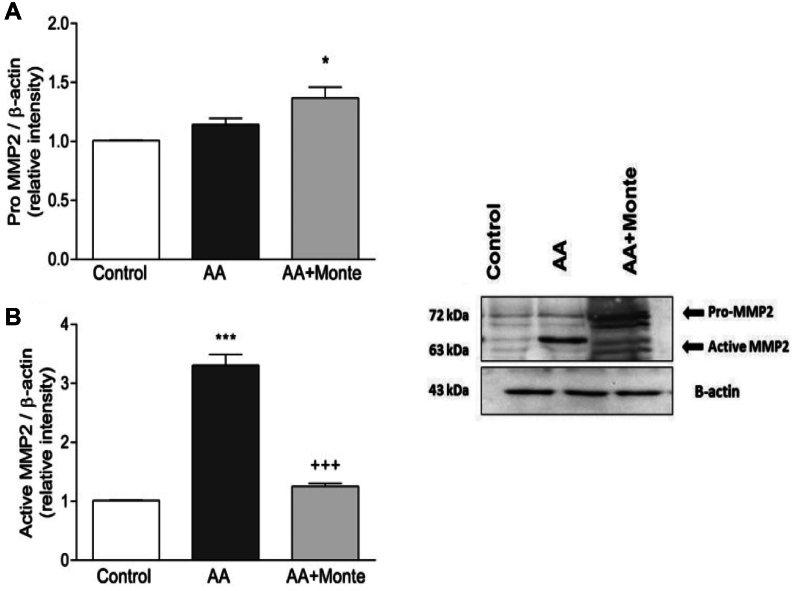
Pro-MMP-2 and active MMP-2 protein expression in aortic tissue. **(A)** Semiquantitative analysis of pro-MMP-2 normalized to β-actin shows increased levels in the AA group compared with controls, with further elevation in the AA + montelukast group (*P* < .05 vs AA). **(B)** Active MMP-2 expression was markedly elevated in the AA group (∗*P* < .001 vs control) and significantly reduced following montelukast treatment (+++*P* < .001 vs AA). The representative Western blot (*right*) displays both the pro-form (pro-MMP-2) and active form of MMP-2, along with β-actin as a loading control. Montelukast administration attenuated the CaCl_2_-induced upregulation of active MMP-2, consistent with its protective effects on aortic remodeling. *AA*, abdominal aortic aneurysm; *MMP*, matrix metalloproteinase.

**Fig 2:**
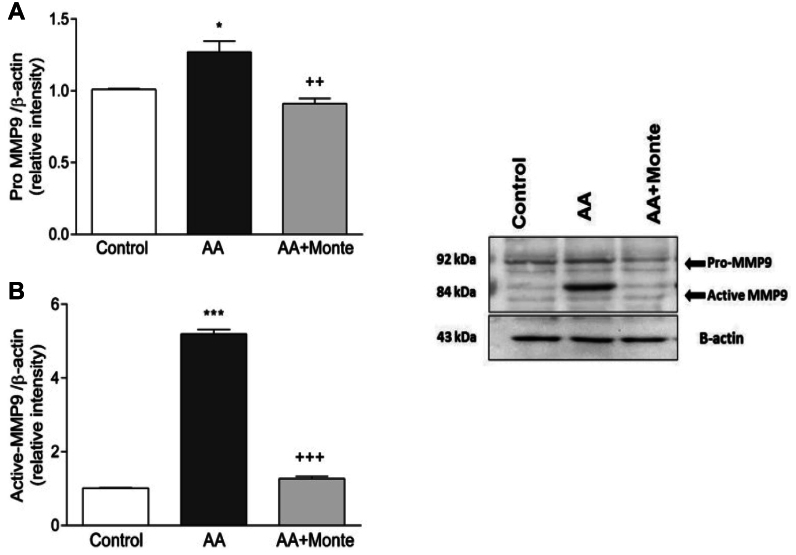
Pro-MMP-9 and active MMP-9 protein expression in aortic tissue. **(A)** Semiquantitative analysis of pro-MMP-9 normalized to β-actin shows a significant increase in the AA group compared with controls (*P* < .05), while montelukast treatment significantly reduced pro-MMP-9 levels (++*P* < .01 vs AA). **(B)** Active MMP-9 expression was markedly elevated in the AA group (∗∗*P* < .001 vs control) and strongly suppressed after montelukast treatment (+++*P* < .001 vs AA). The representative Western blot (*right*) displays both the pro-form (pro-MMP-9) and the active form of MMP-9, with β-actin used as the loading control. Montelukast effectively attenuated CaCl2-induced upregulation of both pro- and active MMP-9, supporting its inhibitory effects on extracellular matrix (ECM)-degrading pathways. *AA*, abdominal aortic aneurysm; *MMP*, matrix metalloproteinase.

Semiquantitative Western blot analysis demonstrated that both MMP-2 and MMP-9 expression levels were significantly elevated in the AA group compared with the sham group (*P* < .001), whereas montelukast treatment markedly reduced the expression of both enzymes (*P* < .001). In addition to the active forms used for quantitative analysis, pro-MMP-2 and pro-MMP-9 bands were also detected in all groups. These pro-form bands exhibited the same directional changes as the active enzymes, showing increased expression in AA rats and attenuation following montelukast treatment, further supporting the suppressive effect of montelukast on MMP upregulation (Figs 1 and 2).

### Tissue 8-hydroxy-2' -deoxyguanosine levels and SOD activity

**Fig 3:**
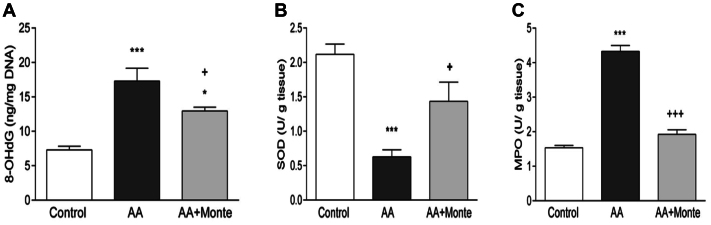
**(A)** Tissue 8-OHdG levels as an indicator of oxidative DNA damage. The AA group showed a marked increase in 8-OHdG concentrations compared with the control group (∗∗*P* < .001), demonstrating significant oxidative DNA injury following CaCl_2_-induced aneurysm formation. Montelukast treatment significantly reduced 8-OHdG levels compared with untreated AA rats (*P* < .05; +*P* < .05). Values are expressed as ng 8-OHdG per mg DNA and presented as mean ± SEM. **(B)** Superoxide dismutase (*SOD*) activity in aortic tissue. SOD activity was markedly reduced in the AA group compared with the control group (∗∗*P* < .001), indicating impaired antioxidant defense following CaCl_2_-induced aneurysm formation. Montelukast treatment significantly restored SOD activity (+*P* < .05 vs AA), reflecting improved antioxidant capacity in treated animals. Data are expressed as U/g tissue and presented as mean ± SEM. **(C)** Myeloperoxidase (*MPO*) activity in aortic tissue. MPO activity was significantly elevated in the AA group compared with the control group (∗∗*P* < .001), indicating pronounced neutrophil-driven inflammatory response after CaCl_2_-induced aneurysm formation. Montelukast treatment markedly reduced MPO activity (+++*P* < .001 vs AA), demonstrating its anti-inflammatory and antioxidant effects. Data are expressed as U/g tissue and presented as mean ± SEM. *AA*, abdominal aortic aneurysm; *MMP*, matrix metalloproteinase; *SEM*, standard error of the mean.

Oxidative DNA damage, indicated by elevated 8-hydroxy-2' -deoxyguanosine (8-OHdG) levels, was significantly higher in the AA group compared with the sham group (*P* < .001). Montelukast administration significantly reduced 8-OHdG levels compared with untreated AA rats (*P* < .05) (Fig 3, *A*).

SOD activity, a marker of antioxidant defense, was significantly decreased in the AA group relative to the sham group (*P* < .001). Treatment with montelukast restored SOD activity to levels significantly higher than those observed in untreated AA rats (*P* < .001) (Fig 3, *B*).

### Aortic MPO activity

MPO activity in aortic tissue was markedly increased in the AA group compared with the sham group (*P* < .001). Montelukast treatment significantly reduced MPO activity compared with untreated AA rats (*P* < .05-.001), indicating potent anti-inflammatory and antioxidant effects (Fig 3, *C*).

### Histological findings

**Fig 4:**
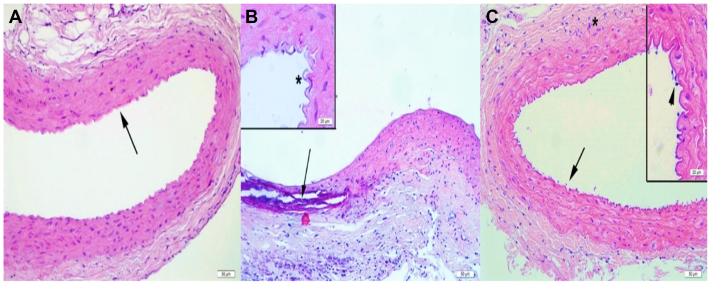
**(A)** Representative histological appearance of the abdominal aorta from the Sham group (hematoxylin and eosin [H&E] staining). The aortic wall demonstrates normal structural organization with intact intima, media, and adventitia layers. The *black arrow* indicates well-preserved elastic lamellae within the medial layer, without evidence of fragmentation or inflammatory cell infiltration. No pathological alterations are observed in the vessel wall. **(B)** Representative histological appearance of the abdominal aorta from the AA group (H&E staining). Severe structural deterioration is evident following CaCl_2_-induced injury. The *black arrow* indicates pronounced fragmentation and disruption of the elastic lamellae within the media, accompanied by intense inflammatory cell infiltration. The asterisk (∗) in the inset marks the irregular and damaged endothelial surface with loss of normal intimal architecture. These findings illustrate the characteristic medial degeneration and inflammatory response observed in untreated aneurysmal aortic tissue. **(C)** Representative histological appearance of the abdominal aorta from the AA + Montelukast group (H&E staining). The aortic wall shows largely preserved elastic lamellae and improved structural organization. The *black arrow* indicates areas with reduced leukocyte infiltration in the media, demonstrating the anti-inflammatory effects of montelukast. The asterisk (∗) marks the adventitial layer, where collagen fiber organization appears restored compared with the untreated AA group. The inset highlights the endothelial surface (*arrowhead*), showing minimal inflammatory cell accumulation and preserved intimal architecture.

**Fig 5:**
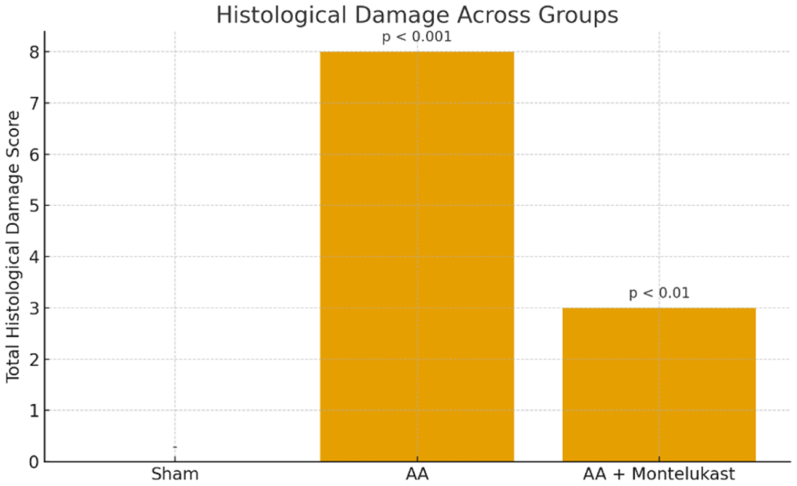
Total histological damage scores across experimental groups. Histological injury was quantified using a semiquantitative scoring system evaluating leukocyte infiltration, elastic lamina disruption, and collagen fiber integrity (0-3 scale for each parameter). The sham group exhibited no detectable histological damage (score = 0). The AA group showed a marked increase in total damage (score = 8; *P* < .001 vs sham), reflecting severe inflammatory infiltration, elastic lamina fragmentation, and collagen disorganization. Montelukast treatment significantly reduced histological injury, yielding a total score of 3 (*P* < .01 vs AA). Data are presented as mean values. *AA*, abdominal aortic aneurysm.

Histological evaluation revealed normal vascular architecture in the sham group, with intact intima, media, and adventitia layers and no detectable inflammatory or structural abnormalities (Fig 4, *A*). In contrast, the AA group exhibited pronounced pathological changes, including dense leukocyte infiltration, marked elastic lamina fragmentation, and collagen fiber disruption, corresponding with a significantly elevated total histological damage score (8; *P* < .001 vs sham) (Fig 4, *B*). Montelukast treatment substantially improved these alterations, as reflected by reduced inflammatory cell infiltration, minimal elastic lamina disruption, and better preservation of collagen structure (Fig 4, *C*), yielding a markedly lower damage score (3; *P* < .01 vs AA) (Fig 5).

## Discussion

Despite significant advances in surgical repair, an effective pharmacological strategy capable of halting AAA progression has yet to be established, underscoring the need for agents that can target early pathogenic mechanisms.^16^ In this context, our findings not only confirm the inhibitory effect of montelukast on MMP expression, but also provide further mechanistic insight by demonstrating concurrent improvements in oxidative and inflammatory pathways. The restoration of SOD activity, together with marked decreases in MPO and 8-OHdG levels, indicates that montelukast interrupts the cycle of oxidative stress and proteolytic activation that contributes to aortic wall degeneration. These biochemical alterations were consistent with histological observations showing improved preservation of elastic and collagen fibers in montelukast-treated animals.

LTs, derived from the 5-lipoxygenase pathway, are potent lipid mediators with key roles in vascular inflammation. CysLTs such as LTC_4_, LTD_4_, and LTE_4_ act via the CysLT_1_ receptor to increase vascular permeability and induce smooth muscle contraction.^22^^,^^23^ Experimental evidence suggests that LTD_4_ can also stimulate MMP expression within the aortic wall.^13^ Montelukast, a selective CysLT_1_ receptor antagonist widely used for asthma, exhibits both anti-inflammatory and antioxidant effects and has been shown to decrease atherosclerotic and aneurysmal vascular injury in experimental settings.^15^^,^^16^ Although montelukast is mechanistically linked to CysLT_1_ receptor blockade, direct measurement of CysLTs or their metabolites was not performed in this study, limiting our ability to confirm upstream pathway modulation. Furthermore, an external positive control model of CysLT production was not included to verify montelukast's pharmacodynamic activity at the administered dose. These limitations restrict definitive confirmation of pathway-specific inhibition and warrant investigation in future studies.

In the present study, montelukast administration significantly attenuated AAA formation in rats subjected to periaortic CaCl_2_ application, a well-established model that mimics human aneurysmal pathology.^17^ Our findings revealed a marked decrease in both MMP-2 and MMP-9 expression, key enzymes involved in ECM degradation and vessel wall weakening.^24^ This outcome is consistent with prior reports in murine and elastase-induced models demonstrating that montelukast suppresses MMP-mediated proteolysis.^7^^,^^8^ Importantly, we also observed parallel decreases in the pro-forms of MMP-2 and MMP-9, which represent the inactive precursors that are converted into active enzymes during ECM breakdown. The simultaneous reduction of both pro-MMP and active MMP bands after montelukast treatment suggests that montelukast exerts an upstream regulatory effect on MMP synthesis and activation pathways, thereby contributing to the preservation of aortic wall integrity.

Beyond confirming these effects on MMP activity, our work extends prior findings by demonstrating montelukast's influence on oxidative and inflammatory pathways in a rat model of AAA. Montelukast treatment restored SOD activity and reduced MPO activity—two critical determinants of redox balance. Elevated MPO activity, known to promote oxidative injury and activate MMPs,^25^ was significantly suppressed after montelukast administration. These results suggest that montelukast mitigates the vicious cycle of oxidative stress and proteolytic activation that drives aneurysm progression. These results support the hypothesis that MPO inhibition is a key mechanism underlying the anti-inflammatory and antioxidant effects of montelukast.

Oxidative stress plays a central role in AAA pathogenesis, as evidenced by elevated levels of 8-OHdG, a marker of oxidative DNA damage.^19^^,^^21^^,^^26^ In this study, montelukast significantly decreased 8-OHdG levels, indicating attenuation of ROS-mediated DNA injury. Additionally, montelukast restored SOD activity, a critical antioxidant defense enzyme whose activity is often decreased in AAA patients. By enhancing SOD activity, montelukast likely decreased ROS accumulation, thereby breaking the cycle of oxidative stress and inflammation.

Our findings showed that CaCl_2_-induced injury resulted in marked infrarenal aortic dilation, whereas montelukast treatment significantly attenuated this enlargement, consistent with its protective effects on aortic structure. However, it should be noted that postmortem aortic diameter measurements were performed without perfusion fixation, and therefore the recorded values may underestimate true in vivo diameters under physiological intraluminal pressure. Despite this methodological limitation, the relative differences between groups remain valid, as all measurements were conducted using the same standardized procedure and under identical conditions.

Histological analyses supported these biochemical outcomes, revealing decreased leukocyte infiltration and improved preservation of elastic and collagen fibers in montelukast-treated rats. Collectively, our findings indicate that montelukast confers vascular protection in a CaCl_2_-induced rat model of AAA through complementary mechanisms, including suppression of MMP activity, reduction of MPO-derived oxidative stress, and restoration of antioxidant capacity. These results highlight montelukast's potential to mitigate both inflammatory and oxidative pathways involved in aneurysm progression.

Montelukast demonstrated notable anti-inflammatory and antioxidant effects in this experimental model and was associated with improved preservation of aortic wall integrity together with reductions in MPO activity and MMP-2/MMP-9 expression. Although these findings support the potential clinical relevance of montelukast as an adjunctive therapeutic strategy for limiting AAA progression, several limitations should be acknowledged. The study used a single dose and treatment duration, and additional work is required to evaluate dose-response relationships, long-term outcomes, and translational applicability in human subjects. Therefore, although the results are promising, further preclinical and clinical investigations are needed before definitive conclusions regarding therapeutic efficacy can be made.

## Conclusions

This study demonstrated that montelukast attenuates the development of AAA in rats subjected to periaortic CaCl_2_ application. The protective effects of montelukast appear to be mediated through the suppression of MMP activity, restoration of SOD levels, and reduction of MPO-driven oxidative injury. By mitigating both inflammatory and oxidative mechanisms, montelukast contributes to the preservation of aortic wall structure.

These findings, consistent with previous murine and elastase-induced models, provide additional mechanistic insight by highlighting the role of montelukast in modulating antioxidant defenses in a rat model. Given its established clinical safety and accessibility, montelukast may represent a promising adjunctive therapy for limiting AAA progression. Further preclinical and translational studies are warranted to confirm these effects and define optimal therapeutic conditions.

## Author Contributions

Conception and design: GT, GS, MK

Analysis and interpretation: GT, OC, SC, GS, MK

Data collection: GT

Writing the article: GT

Critical revision of the article: GT, OC, SC, GS, MK

Final approval of the article: GT, OC, SC, GS, MK

Statistical analysis: OC, GS

Obtained funding: Not applicable

Overall responsibility: GT

## Funding

This work was supported by BAPKO (Coordination Unit for Scientific Research Projects). The sponsor had no role in the study design; collection, analysis, and interpretation of data; manuscript writing; or the decision to submit the manuscript for publication.

## Disclosures

None.
